# Forgetting having denied: The “amnesic” consequences of denial

**DOI:** 10.3758/s13421-017-0781-5

**Published:** 2017-12-20

**Authors:** Henry Otgaar, Tameka Romeo, Niki Ramakers, Mark L. Howe

**Affiliations:** 1 Maastricht University, Maastricht, The Netherlands; 2 City University of London, London, UK; 3 Faculty of Psychology and Neuroscience Section Forensic Psychology, Maastricht University, Maastricht, The Netherlands

**Keywords:** Denial, Memory, Forgetting, Repression, Deception

## Abstract

The concept of denial has its roots in psychoanalysis. Denial has been assumed to be effective in blocking unwanted memories. In two experiments, we report that denial has unique consequences for remembering. In our two experiments, participants viewed a video of a theft, and half of the participants had to deny seeing certain details in the video, whereas the other half had to tell the truth. One day later, all participants were given either a source-monitoring recognition or a recall task. In these tasks, they were instructed to indicate (1) whether they could remember talking about certain details and (2) whether they could recollect seeing those details in the video. In both experiments, we found that denial made participants forget that they had talked about these details, while leaving memory for the video itself unaffected. This denial-induced forgetting was evident for both the source-monitoring recognition and recall tests. Furthermore, when we asked participants after the experiment whether they could still not remember talking about these details, those who had to deny were most likely to report that they had forgotten talking about the details. In contrast to a widely held belief, we show that denial does not impair memory for the experienced stimuli, but that it has a unique ability to undermine memory for what has been talked about.

Memories are not valued equally: We have a strong desire to forget certain experiences because of their distressing characteristics. For example, the death of a close relative might lead to a cascade of emotions that people do not wish to confront. This type of coping style is thought to be an effective way of managing the emergence of unpleasant recollections. One strategy often employed is denial. *Denial* has been theorized as a mental operation to block out painful thoughts and emotions. The concept of denial has its historical roots in psychoanalysis. Regarded as a type of defense mechanism in the psychodynamic school of thought, it refers to a complete refusal to face certain troubling issues. The concept of denial has attracted particular attention in the domain of memory, where it has often been assumed, but not explicitly tested, that “memory may be far more amenable to denial” (Baumeister, Dale, & Sommer, p. 1111). In the present two experiments, we show that denial has the ability to undermine memory in unique and novel ways.

Denial is sometimes used to deal with trauma. For example, it is not uncommon for victims of sexual abuse to resort to using denial to cope by avoiding acknowledgement of the abuse (Jackson, 2006). The general rationale for this avoidance is that victims of sexual abuse find it difficult to talk about their abusive experiences. Many do not disclose any information concerning the traumatic incident(s), and some victims engage in (falsely) denying that they were abused even when the abuse has been documented (e.g., Goodman-Brown, Edelstein, Goodman, Jones, & Gordon, 2003; Lyon, 2007). There are various reasons why victims resort to denial, one of the more obvious ones being shame (Azad & Leander, 2015). Victims may also be externally pressured to deny (e.g., coercion from the perpetrator) (Paine & Hansen, 2002). Importantly, during the course of a well-conducted forensic interview, an initial denial may shift into a full disclosure (e.g., Hershkowitz, Orbach, Lamb, Sternberg, & Horowitz, 2006). Juxtaposed against the fact that there can be significant time delays between abuse’s onset and disclosure, a question arises concerning what the consequences might be of such an initial denial on the quantity and quality of information that is subsequently reported. Hence, it is important to examine what effect denial might exert on memory.

## The effects of denial on memory

Empirical research into the mnemonic effects of denial has been limited. This work has focused mainly on experimenters instructing participants to deny that an event that they experienced occurred, something that is roughly analogous to a perpetrator pressuring their victims to deny the experienced abuse. For example, Vieira and Lane (2013) presented several pictures (e.g., of an apple) to participants. After this, participants received labels of studied and unstudied pictures. Under each label, participants were instructed to repeatedly be truthful by describing the picture or to lie by (falsely) denying that they had seen the picture. Following a delay of two days, a source memory test was provided to participants in which they were asked whether or not they had studied a picture and whether they had lied or told the truth. Participants’ memory for whether they had studied an item was affected by the type of memory condition (truth or lie) they had been assigned to in the rehearsal phase for that item. Specifically, when asked whether they had studied the items previously, participants had greater difficulty remembering whether they had studied an item in the first session if they had later falsely denied having studied it than if they had to tell the truth about the studied item. However, this was not strong evidence that forgetting had occurred, since Vieira and Lane did not find that false denials led to poorer memory than for items that had not been rehearsed.

We have recently shown that denials can affect memory in a unique way (Otgaar, Howe, Memon, & Wang, 2014; Otgaar, Howe, Smeets, & Wang, 2016). In our experiments, participants were presented with some stimuli (e.g., a video), and after this, one group had to deny that certain details had been presented when in fact they had. After a delay (a day or week), participants received a source-monitoring recognition test in which they received the following two questions. First, they were asked whether they had talked about a certain detail during the first session. Second, they were asked whether they could remember seeing that particular detail during the stimulus presentation. As in Vieira and Lane’s (2013) study, memory for the stimuli was unaffected by the act of denial. However, more interestingly, in the false-denial group, participants were more likely to report that they had not talked about a certain detail during the first session, when in fact they had. In a sense, they “forgot” that they had denied seeing a certain detail during the first session. This effect has been called *denial-induced forgetting*.

What makes this effect so intriguing and different is that in contrast to other forgetting phenomena, such as directed forgetting or retrieval-induced forgetting, the present effect is not related to the forgetting of encoded stimuli. That is, denial-induced forgetting pertains to an effect in which participants are unable to recollect that they talked about a certain detail during the first interview session. This implies that the act of denial adversely impacts recollection of that denial at the very time the denial is being issued. Also, the effect seems to be robust, since it has been shown in children and adults (Otgaar, Howe, et al., 2014), using different stimuli (pictures and videos; Otgaar et al., 2016), using different retention intervals (one day and one week; Otgaar, Howe, et al., 2014; Otgaar et al., 2016), and for neutral and emotionally negative stimuli (Otgaar et al., 2016). Furthermore, previous experimentation has ruled out the possibility that the effect was caused by a lack of confidence or processing of the items at the time of the denial (Otgaar et al., 2016).

From a practical perspective, the phenomenon of denial-induced forgetting might be relevant in the context of the reliability of the memory of victims (and eyewitnesses). That is, victims of abuse are sometimes interviewed repeatedly. When, during the first interview, they (falsely) deny having experienced certain abuse-related details and then during a subsequent interview “forget” what they talked about during the first interview, their statements might appear inconsistent. Why this is relevant is because legal professionals often falsely assume that such inconsistent statements are unreliable, and as such, victims might have the appearance of being unreliable. The consequence could ultimately be that their statements will not be taken seriously in the courtroom (e.g., Smeets, Candel, & Merckelbach, 2004).

What we do not know at present is the extent of this phenomenon and whether denial-induced forgetting can be detected in other memory tasks. That is, previous research has employed a rather simple source-monitoring test. Specifically, previous participants had to recognize whether they remembered certain stimuli and whether they had also talked about these stimuli. From a purely theoretical stance, it is relevant to assess whether this effect can be generalized to other memory processes such as recall. The relationship between recall and recognition has a long history in psychology (e.g., J. R. Anderson & Bower, 1972, 1974; Gillund & Shiffrin, 1984; Haist, Shimamura, & Squire, 1992). The general finding is that recognition is easier than and superior to recall. Indeed, traditional theories postulated that recall requires more memory strength for a response than does recognition (McDougall, 1904) and that recognition is often based purely on familiarity judgments (J. R. Anderson & Bower, 1973). Following these theoretical principles, the basic tenet became that recall entails more extensive reinstatement of an encoded event than does recognition (e.g., Craik, 2002; Lockhart, Craik, & Jacoby, 1976).

So, a more stringent test of whether denial leads to forgetting would be to investigate the effects of denial on both recall *and* recognition. If denial has a strong adverse impact on memory, it would lead to memory undermining effects in recall, as well. Examining this is all the more relevant because certain memory phenomena are found only for recognition and not for recall. For example, a consistent pattern in the false-memory literature is that false recognition is evoked more easily for emotionally negative than for neutral material. This pattern, however, is not found for recall (Howe, Candel, Otgaar, Malone, & Wimmer, 2010). On the other hand, certain robust memory phenomena, such as retrieval-induced forgetting, are found for both recall and recognition (e.g., Gómez-Ariza, Lechuga, Pelegrina, & Bajo, 2005).

The examination of the effect of denial on memory recall and recognition is also relevant from a theoretical perspective. Recently, a memory-and-deception (MAD) model has been proposed to explain the effects of different types of deception on memory (Otgaar & Baker, 2018). According to MAD, denial can be seen as a form of deception. Specifically, MAD proposes that the memory deterioration effect of denial is caused by the fact that the act of denial monopolizes cognitive resources. Because of this, participants are less likely to rehearse what they have denied, leading to forgetting effects. If true, the lack of rehearsal would lead to forgetting effects not only in recognition tasks, but also in recall tasks. Also, from a practical perspective, examining denial-induced forgetting in recall tasks is warranted. For example, in many interview settings, victims who initially deny that an event has taken place are frequently given follow-up, open-ended questions that target recall memory (Lamb et al., 2007).

## The present experiments

In the present experiments, participants had to view a video of a theft, and half of the participants had to deny that they saw certain details present in the video. One day later, half of the participants received the usual source-monitoring task, whereas the other half received a recall task. In the recall task, the participants were instructed to recall as many details as they could that they had talked about during the first session. Furthermore, they were asked to recall as many details as they could from the video. As an exploratory aim, participants were also asked to rate their belief in, and memory for, items that were mentioned during the memory tests (Otgaar, Scoboria, & Mazzoni, 2014). Our main prediction was that denial-induced forgetting would be found for both the source-monitoring recognition and recall tasks.

## Experiment 1

### Method

#### Participants

On the basis of an a priori power analysis (G*Power; Faul, Erdfelder, Lang, & Buchner, 2007) with a power of .80 and a medium effect size for the difference between the false-denial and control groups (*f* = 0.25), a total sample size of 90 was needed. The present study used a total sample size of 87 (mean age = 22.38, *SD* = 5.98, range 17–59; 69 women, 18 men). Participants were recruited by means of advertisements posted at Maastricht University. After participating, each person received compensation for their time (e.g., participant points or a voucher).

#### Materials

##### Video

We used a video that has been used in our previous work (Otgaar, Howe, et al., 2014; Otgaar et al., 2016) as well as in other memory studies (Takarangi, Parker, & Garry, 2006). The video is called “Eric the Electrician.” In the video, Eric enters a house and steals several items (e.g., jewelry, CD) in that house. The duration of the video is 6.5 min.^1^

#### Design and procedure

We used a between-subjects design in which participants were randomly assigned to one of four conditions (denial–source monitoring, denial–free recall, control–source monitoring, and control–free recall). The present experiment comprised two sessions separated by a one-day interval. The first session contained two testing moments, and the second session contained one testing moment. In the control group, participants were asked to answer the questions that were asked as honestly as possible. During the second session, half of the participants were asked to take part in a source-monitoring recognition task (yes/no questions), and half of the participants were asked to take part in a free-recall task. In the denial group, participants were asked to (falsely) deny having seen certain details in the video during the first session. For instance, when asked what vehicle Eric arrived with, the participant was required to say that Eric did not arrive with a vehicle. In the second session, the same partition was made as in the control condition: Half of the participants took part in a source-monitoring recognition task (yes/no questions), and the other half was asked to take part in a free-recall task.

##### Session 1

All participants first viewed a video of Eric the electrician, after which they engaged in the first distractor task (Tetris, to prevent rehearsal of the information) for 5 min. They then completed a memory test that contained ten items about details that had been present in the video (true questions: e.g., “Where did Eric find the key”?). After each item, participants had to indicate their memory and belief for the item (belief: 1 = *definitely did not happen*, 8 = *definitely did happen*; memory: 1 = *no memory at all*, 8 = *clear and complete memory*; Scoboria, Mazzoni, Kirsch, & Relyea, 2004). Then they engaged in the second distractor task (Bubble Shooter) for 5 min. Following this, participants received a second memory test. Specifically, the participants in the control conditions were instructed to answer honestly and without guessing (e.g., “What kind of vehicle did Eric arrive with?” Correct response: a van), and the participants in the denial conditions were instructed to deny in response to every question (e.g., “What kind of vehicle did Eric arrive with?” Correct response: Eric did not arrive with a vehicle). This task consisted of 12 questions, of which eight were true (and were also asked on the first memory test) and four concerned details that were not shown in the video.^2^ Importantly, the participants in the denial condition were corrected if they did not provide a denial as instructed. For example, if a participant stated “Eric did not arrive with anything,” he or she was told how to deny by using an example, to make sure every participant came up with the same denial (e.g., “Eric did not arrive with a vehicle”).

##### Session 2

One day later, the participants in the source-monitoring conditions were asked to complete the source-monitoring recognition task. During this task, participants were asked to answer 16 items (ten true—five apiece asked about the first and second memory tests—and six false) that each contained two questions. The first question referred to whether the participant had talked about these details during the interviews in Session 1. The second question referred to whether the participant had seen certain details during the video. Both questions should be answered with a “yes” or “no.” *True* details referred to details that had been present in the video, whereas *false* details had not been present in the video. After each item, participants were asked to indicate their memory and belief for the item. Participants in the free recall conditions were asked to write down as many details as they could remember from the interview first, and then from the video. They also had to indicate their memory and belief strength (belief: 1 = *definitely did not happen*, 8 = *definitely did happen*; memory: 1 = *no memory at all*, 8 = *clear and complete memory*) for every detail they wrote down. At the end of the experiment, all participants received a debriefing.

### Results and discussion

#### Baseline memory performance

To examine whether participants were able to accurately recollect details from the video, we calculated the overall mean baseline memory performance across groups. The overall mean proportion baseline memory performance across groups was .76 (*SD* = .16), suggesting that the video was not too difficult and complex for participants. To examine whether the various groups differed in terms of their memory performance before half of the participants had to deny some details, a one-way analysis of variance (ANOVA) was conducted on the performance on the first memory test. The groups did not differ statistically in terms of memory performance, *F*(3, 83) = 0.49, *p* = .69, *η*_p_^2^ = .02.

#### Memory effects of denial

**Fig. 1:**
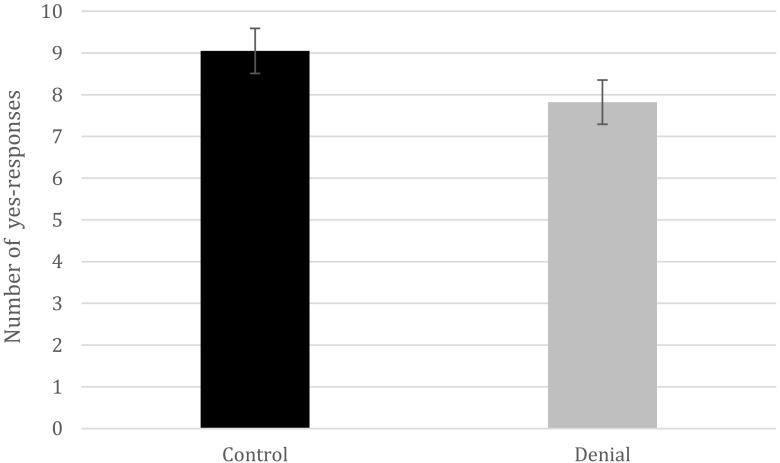
Experiment 1: Raw numbers of “yes” responses that participants provided on the source-monitoring recognition task between the denial and control groups (error bars represent 95% confidence intervals).

The most important analyses focused on whether the act of denial can lead to memory-undermining effects for both (source-monitoring) recognition and recall. Importantly, because recall and recognition are measured differently, we used separate independent-samples *t* tests to investigate the effects of denial on recognition and recall. To start, we analyzed the effect of denial on the recognition of details that were talked about during the memory tests. As expected, we found evidence for a denial-induced forgetting effect, *t*(41) = 3.27, *p* = .002, *d* = 0.99, 95% CI [0.05, 0.99]. That is, the participants in the denial group (*M*_prop_ = .78, *SD* = .09) were less likely to remember that they had talked about details presented during the memory tests, when in fact they had, than were the participants in the control group (*M*_*prop*_ = _*.*_91, *SD* = .15; Fig. 1). To examine whether our denial-induced forgetting effect would also appear using alternative statistical tests, we conducted a Bayesian analysis. A Bayes factor_10_ of 16.50 with a prior of 0.71 was also found, which means that our data were more in favor of the alternative hypothesis (= a difference between the two groups) than the null hypothesis (= no difference between the groups). We performed the same analyses on false details that were mentioned during the interviews. No statistical differences emerged between the two groups, *t*(42) = – 1.78, *p* = .08. We also looked at whether denying seeing details affected memory for the video. No statistical difference was found between the two groups when looking at the presented details, *t*(41) = – 0.73, *p* = .47. Similarly, no effect was likewise found when focusing on the false details, *t*(41) = – 0.97, *p* = .34.

The next question was whether a denial-induced forgetting effect would be found when looking at the recall data. When focusing on the average number of details that were discussed during Session 1, we found some support for the denial-induced effect for false details, *t*(35.01) = 3.03, *p* = .005, in that the denial group (*M* = 0.52, *SD* = 0.60) forgot having denied these details the day before more than did the control group (*M* = 1.27, *SD* = 0.98). A Bayes factor_10_ of 8.93 with a prior of 0.71 was found for this result, which indicates that our data are again more in favor of the alternative than of the null hypothesis. However, no effect of denial on true recall was observed, *t*(41) = – 0.60, *p* = .55. Analyses on the recall data for details that had been seen in the video revealed a consistent pattern. No denial-induced forgetting was revealed for either true, *t*(41) = – 0.96, *p* = .34, or false, *t*(41) = – 0.23, *p* = .82, recall.

#### Exploratory analyses

##### Belief and memory

We also conducted some exploratory analyses on the belief and memory rating data. A one-way ANOVA on the belief and memory ratings between the denial and control groups for the first memory test did not reveal any statistically significant effects [belief: *F*(3, 83) = 1.35, *p* = .26; memory: *F*(3, 83) = 0.79, *p* = .50]. For items addressed on the source-monitoring test, no statistical difference emerged between the denial and control groups for all measures (i.e., true, false items) for either belief or memory, *t*s < 1.21. For the recall data, again no statistical differences emerged for either the belief or the memory data, *t*s < 2.00.

##### Issues with recall data

On the basis of the analyses described so far, one might conclude that we replicated the denial-induced forgetting effect for source-monitoring recognition and also found evidence for this effect in the recall data. However, in retrospect we realized that the groups that received a recall task differed in many ways from the recognition groups. First, the participants who were presented with the source-monitoring recognition task received the questions orally, but the participants who received the recall task had to write the items that they could still recollect. Second, and more importantly, the participants who received the recall task were not given any time limit, whereas those presented with the source-monitoring recognition task had a fixed-duration task. This second difference was particularly problematic because the participants in the recall groups had more time to think about all of the items, and this could have masked any potential recall difference between the denial and control groups.

Indeed, when we looked at the distribution of the recall data, we saw that 41.8% (*n* = 18) of the participants came up with details in the second half of their recall attempt, but only 9.3% (*n* = 4) recollected details in the first half. This implies that the additional time resulted in most participants recollecting the details toward the end. Hence, to remedy this issue and the issue that the recall and source-monitoring recognition tasks were administered differently, we conducted a second experiment in which we improved our methodology. That is, the participants in both the recall and source-monitoring recognition tasks had to complete the tasks by writing down their responses during Session 2. Furthermore, in Experiment 2 a time limit was introduced for the memory tests.

## Experiment 2

Experiment 2 was identical to Experiment 1 (i.e., similar design and material), except for what happened during Session 2.

### Method

#### Participants

On the basis of an a priori power analysis (G*Power; Faul et al., 2007) with a power of .80 and a medium effect size for the difference between the denial and control groups (*f* = 0.25), a total sample size of 90 was needed. In Experiment 2, 100 participants (mean age = 21.23, *SD* = 3.63, range 18–44; 89 women, 11 men) were tested. As in Experiment 1, participants were acquired by means of advertisement at Maastricht University. After participating, they received a compensation for their time (e.g., participant points or a voucher).

#### Design and procedure

The difference between Experiments 1 and 2 occurred during Session 2. That is, in this experiment we imposed a time limit during the recall and recognition tasks. On the second day of the study (24 h later), the participants in the source-monitoring conditions were asked to complete the source-monitoring task themselves. During this task, participants were asked to complete the task using the same items and questions as in Experiment 1. Furthermore, they were not only asked whether they were able to remember whether they had seen a certain detail in the video, they also had to come up with that detail. For instance, when participants were asked the question “When watching the video, did you see what vehicle Eric arrived with?,” participants were expected to answer with “yes,” but they were also expected to write down the vehicle (in this case, “a van”). Also, participants had to indicate their memory and belief about the detail the question referred to (belief: 1 = *definitely did not happen*, 8 = *definitely did happen*; memory: 1 = *no memory at all*, 8 = *clear and complete memory*).

The participants in the free-recall conditions received the same task as in Experiment 1. However, a time limit of 7.5 min was now used in the free-recall conditions. The research assistant of this experiment (third author) estimated the time that was needed to complete the source monitoring, and this time limit was used for the recall task as well. This time limit was added in order to make the free-recall and source-monitoring conditions more alike with regard to the time needed to complete the task. After this task, some additional exit interview questions were directly posed. These questions concerned the details that had been remembered and talked about during the first session, but that had not been reported in the second session. The experimenter confronted the participants with the fact that they had not reported a certain detail that they had talked about the day before. The experimenter then asked them whether they really did not remember talking about this particular detail. If the participant stated that he or she did not remember the detail, the experimenter asked why the participant thought he or she did not remember talking about that detail. At the end of the experiment, all participants received a debriefing.

### Results and discussion

#### Baseline memory performance

The overall mean proportion baseline memory performance across groups was .77 (*SD* = .15). As in Experiment 1, the groups did not differ statistically in terms of their initial memory performance, *F*(3, 96) = 1.27, *p* = .29, *η*_p_^2^ = .04.

#### Memory effects of denial

**Fig. 2:**
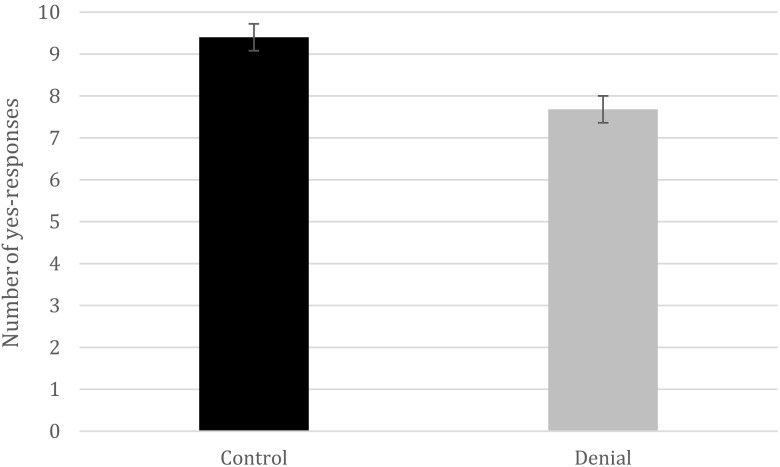
Experiment 2: Raw numbers of “yes” responses that participants provided on the source-monitoring recognition task between the denial and control groups (error bars represent 95% confidence intervals).

**Fig. 3:**
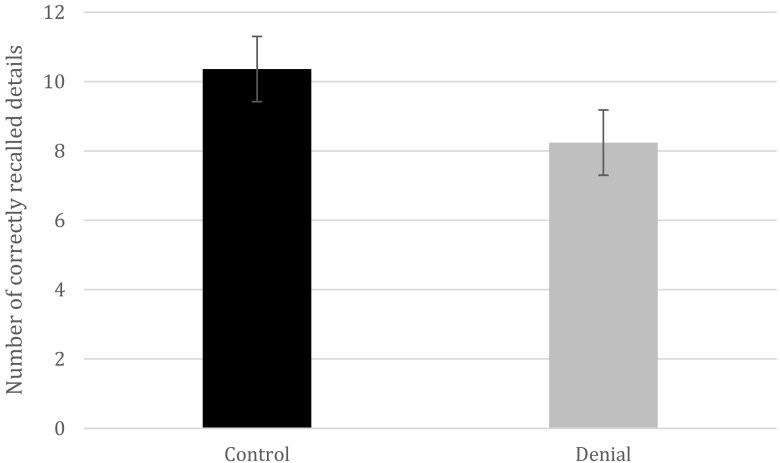
Experiment 2: Numbers of correctly recalled details between the denial and control groups (error bars represent 95% confidence intervals).

Again, we found support for a denial-induced forgetting effect for true details, *t*(48) = 7.77, *p* < .001, *d* = 2.20, 95%CI [0.13, 0.22], with the experimental group (*M*_prop_ = .77, *SD* = .09) denying having talked about certain details more than the control group did (*M*_prop_ = .94, *SD* = .071) (Fig. 2). A Bayes factor_10_ of 12.84 with a prior of 0.71 was also detected. As in Experiment 1, this effect did not appear for the false details, *t*(48) = 0.62, *p* = .54. For the recall data, a denial-induced forgetting effect was detected as well for the true details, *t*(48) = 3.19, *p* = .002, *d* = 0.90, 95%CI [0.79, 3.45], Bayes factor_10_ = 14.70. Specifically, the participants in the denial group (*M* = 8.24, *SD* = 2.09) recalled fewer details that had been talked about than did the participants in the control group (*M* = 10.36, *SD* = 2.58; Fig. 3). This effect also emerged for the false details, *t*(48) = 5.96, *p* < .001, *d* = 1.69, 95%CI [1.06, 2.14], Bayes factor_10_ = 39,880 (control group: *M* = 1.92, *SD* = 1.22; denial group: *M* = 0.32, *SD* = 0.56). Again, no effect of denial was found on memory for the video for either true, *t*(48) = 0.88, *p* = .38, or false, *t*(48) = – 0.22, *p* = .83, details.

#### Exploratory analyses

##### Belief and memory

A one-way ANOVA on the belief and recollection data for the first memory test between the different groups did not demonstrate any statistically significant effects [belief: *F*(3, 96) = 0.88, *p* = .46; memory: *F*(3, 96) = 0.75, *p* = .53]. For items addressed on the source-monitoring and recall tests, no statistical differences emerged between the denial and control groups for all measures (i.e., true items [video and interview], false items [video and interview]) for either belief or memory, *t*s < 1.34.

##### Exit interview

To explore whether participants really did not remember talking about certain details, even when told that they had reported a detail during the first session, we conducted an analysis on the responses of participants directly after the experiment. The answers were in response to questions about the details that had been remembered and talked about during the first session, but that had not been reported in the second session. Participants’ responses were categorized into four groups: 1 = *no answer*, 2 = *remembers it again*, 3 = *does not remember it again*, 4 = *vague memory*. A Fisher’s exact test was performed on the distribution of these group scores. We found a statistically significant effect (Fisher exact test = 80.98, *p* < .001, Cramér’s *V* = .49), showing that the control group was more likely to give no answer (*n* = 46), whereas the participants in the denial group were more likely to say that they could not remember the detail anymore (*n* = 30). This suggests that even when the participants were confronted with the fact they had talked about certain details during the first session, they responded that they could not remember it anymore. Together with the aforementioned denial-induced forgetting results, this provides additional evidence that denial leads to the forgetting of material that has been discussed.

## General discussion

It has been well-documented that people sometimes employ the strategy of denial in order to avoid the acknowledgment of unpleasant issues or experiences. By doing so, unwanted memories are assumed to be less accessible, and hence not as likely to be retrieved. In the present line of investigation, we tested this purported effect of denial on memory performance. We found strong support that denial leads to a special form of forgetting. Specifically, in two experiments we showed that denial did not make people forget the details that they had experienced, but it *did* make them forget that they had talked about certain details. This denial-induced forgetting effect was demonstrated for both source-monitoring recognition and recall tests.

In both experiments, participants viewed a video of a theft and were asked several questions related to the video. After this, half of the participants had to deny that they had seen certain details, whereas the other half had to tell the truth. One day later, participants were asked about their memory for details of the video and about their memory for details that had been discussed during the interview. Half of the participants were involved in a source-monitoring recognition test, whereas the others received a recall task. In both experiments we found that when asked about their memory of the interview, the denial group forgot having talked about these details, whereas in fact, they had. Furthermore, this forgetting effect was only directed at the memory for the interview, since memory for the video was unaffected by the act of denial. Equally interesting, in most of the analyses in which we have found denial-induced forgetting (*n* = 3, 60%), we found that this denial-induced forgetting occurred especially for true details. This could mean the following. First, it shows that demand characteristics are unlikely to have played a role, since one could argue that denial-induced forgetting should then have been present for all types of details (true and false). Second, this result might imply that participants were aware that the false details were false, thereby making them more distinctive. If so, it might be that because of the increased distinctiveness of false details, it would be difficult to create memory-undermining effects by means of denying them. Of course, this is an issue that awaits future empirical scrutiny.

What makes this forgetting effect so remarkable is that it deviates from other well-known forgetting effects in the memory literature, such as retrieval-induced forgetting, directed forgetting, and retrieval suppression (e.g., Hu, Bergström, Gagnepain, & Anderson, 2017; MacLeod, 1999; Murayama, Miyatsu, Buchli, & Storm, 2014). For the latter effects, people are instructed to forget certain stimuli or to “not think” about certain material. The standard finding is that memory for these stimuli is impaired as a result of the instructions. This, however, is not what happens during denial-induced forgetting. Memory for the stimuli is not affected, but memory for the details about denying that these stimuli were seen earlier (as was discussed during a subsequent interview) is compromised. As far as we know, such a forgetting effect is quite unique in the memory literature.

The only forgetting effect that seems to be roughly related to our denial-induced forgetting effect is the forget-it-all-along effect (Arnold & Lindsay, 2002; Schooler, Bendiksen, & Ambadar, 1997). The latter effect refers to the finding that people forget prior instances of remembering when past events are remembered in different ways on separate occasions. Although there are similarities between the two effects, in denial-induced forgetting, people forget having denied and do not forget having remembered. Of course, similar mechanisms might play a role here, and this would be an interesting area for future research.

Another impetus of the present experiments was to assess whether the act of denial led to forgetting in different memory tasks. Previous studies have only focused on the effect of denial on source monitoring (Otgaar, Howe, et al., 2014; Otgaar et al., 2016). In the present experiments, we included two ways to test memory—namely, by using a source-monitoring recognition and a free-recall task. Our reasoning was that denial would only have strong memory-undermining effects if such effects were observed in both memory tests. What we found was that denial-induced forgetting occurred on both memory tasks. Although in Experiment 1 this forgetting effect was not that straightforward for the recall data, we argued that the groups that received the source-monitoring recognition or recall tasks differed in many aspects (e.g., in the time allotted for retrieval), and that this could have affected the results. When-we corrected these issues, we obtained clear evidence that denial undermined memory in both the source monitoring recognition and recall tasks. This finding is so important because recall has been shown to be more effortful, and hence more demanding, than recognition (J. R. Anderson & Bower, 1972, 1974; Gillund & Shiffrin, 1984; Haist et al., 1992). Finding that denial impairs both source-monitoring recognition and recall strengthens the idea that denial can result in forgetting. Alternatively, when we imposed a time limit on the participants in Experiment 2, no forgetting took place, but participants had less time to retrieve the items that had been memorized. However, when we asked participants whether they truly did not remember talking about these details, the participants in the denial group were most likely to indicate that they still could not recollect talking about the details. All in all, this strengthens our idea that denial leads to actual forgetting. Of course, this exit interview happened directly after the memory test; hence, it is unclear whether this forgetting effect would also occur after a delay. Prospective research might dig into this issue.

Inhibition has been proposed to underlie the memory deterioration effects found in retrieval suppression or retrieval-induced forgetting (M. C. Anderson & Green, 2001; Murayama et al., 2014). The idea behind this hypothesis is that inhibitory control mechanisms prevent memories that are unwanted from popping up during retrieval. It is likely that such inhibitory control also occurs during denial-induced forgetting. In the present experiments, when people were instructed to deny having seen certain details, the act of denial of those particular details might have inhibited the memory of talking about those details during the interview. As a result, when participants had to remember talking about those details, the participants who had to deny were less able to retrieve the memory of the interview than were the control participants.

An equally interesting explanation would be to regard denial as a simple form of deception. That is, when participants were instructed to deny, most of the time they were denying seeing certain true details, which can be seen as lying. This is important to note, because lying requires cognitive resources (Otgaar & Baker, 2018; Vrij & Heaven, 1999; Walczyk, Harris, Duck, & Mulay, 2014). If this reasoning is applied to the present experiments, the result is that when participants had to deny a detail, fewer resources were available to memorize the details that had been talked about during the interview. Therefore, the participants in the denial group had impaired memory for the “denial” interview at a follow-up interview. This interpretation would be well in line with the MAD framework, which suggests that the act of deception engages cognitive resources and that the use of such resources might affect memory performance (Otgaar & Baker, 2018).

Alternatively, our results parallel research on the mnemonic effects of feigning amnesia. In this line of research, participants are involved in a mock crime, and one group has to feign amnesia for the crime. What has been found in these studies is that when instructed to tell the truth, the feigners omit details that they experienced, relative to nonfeigners (e.g., van Oorsouw & Merckelbach, 2006). These effects have been interpreted in terms of a lack of rehearsal when participants feign amnesia. Specifically, the rationale is that when simulating amnesia, participants rehearse the experienced event less well and less efficiently, leading to memory-undermining effects of the experienced event. The same might occur during denial-induced forgetting. Of course, the present experiments were designed to examine whether denial truly leads to some form of forgetting, and the goal was not to assess the mechanisms behind denial-induced forgetting. Future experiments could examine this proposed mechanism of cognitive resources by adding an extra group in which resources are depleted as well, but for reasons other than denial (e.g., a divided-attention group).

Denial has often been conceptualized as a strategy to deal with unpleasant experiences, thereby making traumatic memories less likely to be accessed. In this regard, denial has its roots in psychoanalysis and has much overlap with the repression (Baumeister, Dale, & Sommer, 1998). Although our results have shown that denial undermines memory, they should by no means be interpreted as evidence for denial being an efficient means to block out (traumatic) memories. Indeed, we found no evidence whatsoever that denial impacted the recollection of the stimuli themselves. So, in line with previous critiques of the use of repression (e.g., Lindsay & Read, 1994; Loftus, 1994), the present studies add that it is unlikely that denial can be used to suppress traumatic memories. Instead, our studies illustrate a different role for the effects of denial on memory—namely, that it affects recollection of the denial itself, but not of the stimulus that was the target of the denial. Of course, it must be noted here that the stimuli used in the present experiments are a far cry from the traumatic experiences that child abuse victims face, and hence, an empirical question is whether our results would be reproduced when using more negative stimuli than the ones that have been used here and in previous work (Otgaar et al., 2016).

To summarize, in the present experiments we examined the effects of denial on memory. In two experiments, we showed that denial impaired memory on source-monitoring recognition and recall tests. Specifically, we found that denial made participants forget having talked about denying the presence of certain details seen in an earlier video. Since our findings did not show any effects of denial on the memory for the stimuli themselves, denial seems to lead to a unique form of forgetting in both recall and recognition, which to our knowledge has not been previously demonstrated in the psychological literature.

## Footnotes

1 All materials and data sets (analyses and syntax) can be found at https://osf.io/vy5tm/.

2 Our experiment used a procedure similar to that in previous work on denial-induced forgetting. Because in the first study on this effect (Otgaar, Howe, et al., 2014) we were also interested in false memories, false details were added in the memory tests. Hence, these false details were also present in the present experiments.
